# The French Memorial of Edith Cavell

**Published:** 1920-06-19

**Authors:** 


### The French Memorial of Edith Cavell.

LORD BURNHAM'S SPEECH.

In Paris, on June 12, in the Tuileries Gardens, the ceremony described on p. 307 of this issue took place. Viscount Burnham, who delivered his speech in French, said:

I am much honoured by the invitation to take part in this fine act of dedication, this payment of the debt of honour and admiration to a woman who for all time will be transfigured into the personification of heroism and martyrdom among women not only of our time, but of all time. I do it with the greater satisfaction because for twenty years I have been one of the Committee of the London Hospital, to which Nurse Cavell belonged, both by her training and profession. Her memory will be our proudest possession and our finest appeal.

She was not distinguished in her hospital career from the multitude of good women who devote their lives to the care of the sick poor and the healing of those that suffer. She was just the true type of the London Hospital nurse. The crisis of her fate and of the fate of humanity at large brought out the radiant and tempered metal of her character. She had in its highest degree that sense of duty which we British proudly claim as a general attribute of our race, even more than that love of our island home. When she was tried in the fiery furnace by German brutality and revenge, she passed through as if she had been treading a hospital ward. Mr. Brand Whitlock, the American Minister at Brussels, has told the glorious story as well as it can be told, and the journal with which I am associated had the good fortune to give it to the British public. He asks, "Why was Miss Cavell singled out among all the others as the one to be shot at dawn?" and he answers, "Because Edith Cavell was English—that was her offence." Miss Cavell, in her farewell letter to the nurses who were at once her assistants and her comrades, with a humility and a dignity that make her words shine as in letters of gold, wrote:

"S'il y a une de vous qui a un grief contre moi, je vous prie de me le pardonner; j'ai été peut-être quelquefois trop sévère mais jamais volontiers injuste, et je vous ai aimées, toutes beaucoup plus que vous ne croyez.'

It has been well said that those "few lines she wrote, and the simple words she spoke as she was about to die will remain to reveal the heights that human nature may attain, and to sanctify a memory that will be revered as long as faith and honour are revered by men." She suffered because she was an Englishwoman, but she suffered for France as well as for England. French soldiers owe their life and safety to her, because she only knew them as comrades-in-arms for the greatest of all crusades against the dominion of darkness and despair. The days and the years pass, and we all travel along a broken and war-worn road; but so long as human memory endures Nurse Cavell will be among the saints, and the halo of pure mercy and loving kindness will be around her head.

The newspaper Press has become a universal power beyond all powers, because public opinion is the true ruler of the universe, and the Press is the universal echo of public opinion. Tyranny, whatever colours it bears, cannot exist side by side with a free Press. Above all, a free Press is the general interpreter which explains one nation to another nation, each to all and all to each. We exchange to-day the vows of friendship. Here to-day the British Press, for which I speak, welcomes and acclaims this noble act of solidarity and remembrance which crowns our entwined flags and knits together in close affection French and British journalism. I do not forget that I am standing on ground that is soaked with all the romance and much of the glory of French history. To use the words of a pleasant writer, Georges Cain: "Ce jardin où se déroulèrent tant de faits glorieux ou tragiques de votre histoire nationale; et une nuit d'incendie anéantit trois siècles d'art." This adds the charm of an appropriate setting to this beautiful monument. I thank you in the name of the London Hospital; I thank you in the name of the British Press Conference of which I am the president; I thank you in the name of the British people as a member of the British Parliament.

